# The effect of transforming growth factor-β1 on nasopharyngeal carcinoma cells: insensitive to cell growth but functional to TGF-β/Smad pathway

**DOI:** 10.1186/1756-9966-29-35

**Published:** 2010-04-23

**Authors:** Jian Xiao, Qi Xiang, Ye-Chen Xiao, Zhi-Jian Su, Zhi-Feng Huang, Qi-Hao Zhang, Yi Tan, Xiao-Kun Li, Ya-Dong Huang

**Affiliations:** 1 Biopharmaceutical Research and Development Center, Jinan University, Guangzhou, 510632, China; 2 School of Pharmacy, Wenzhou Medical College, Wenzhou 325035, China

## Abstract

**Objectives:**

This study explored the response of nasopharyngeal carcinoma cells to TGF-β1-induced growth suppression and investigated the roles of the TGF-β/Smad signaling pathway in nasopharyngeal carcinoma cells.

**Methods:**

The cells of nasopharyngeal carcinoma cell line CNE2 were treated with TGF-β1. The growth responses of CNE2 cells were analyzed by MTT assay. The mRNA expression and protein subcellular localization of the TGF-β/Smad signaling components in the CNE2 were determined by real time RT-PCR and immunocytochemical analysis.

**Results:**

We found that the growth of CNE2 cells was not suppressed by TGF-β1. The signaling proteins TβRII, Smad 7 were expressed normally, while Smad2, Smad3, and Smad4 increased significantly at the mRNA level. TGF-β type II receptor and Smad7 had no change compared to the normal nasopharyngeal epithelial cells. In addition, Smad2 was phosphorylated to pSmad2, and the activated pSmad2 translocated into the nucleus from the cytoplasm, while the inhibitory Smad-Smad7 translocated from the nucleus to the cytoplasm after TGF-β1 stimulation.

**Conclusion:**

The results suggested that CNE2 cells are not sensitive to growth suppression by TGF-β1, but the TGF-β/Smad signaling transduction is functional. Further work is needed to address a more detailed spectrum of the TGF-β/Smad signaling pathway in CNE2 cells.

## Introduction

Nasopharyngeal carcinoma (NPC) is an epithelial malignancy arising from the mucosal epithelium of the nasopharynx and has a high incidence of metastasis [1]. NPC is classified by the WHO into three histological types: keratinizing squamous cell carcinoma (type I); nonkeratinizing carcinoma, characterized as differentiated (type II); or, undifferentiated (type III) [2]. Although NPC is a rare malignancy in most parts of the world, it is endemic in a few well-defined populations such as the natives in southeast Asia [3], and the incidence of NPC reported in southeast Asia is nearly 20-60 times higher than that reported in the Western countries [4,5].

Development of NPCs are not well understood, the distinctive racial/ethnic and geographic distribution of NPC worldwide suggest that both genetic traits and environmental factors contribute to its development. Investigation of the molecular mechanisms could help illuminate the causes and ultimately the prevention of this remarkable disease. There have been scanty but emerging reports on the importance of cytokines and growth factors in NPC, where most of these investigations have attempted to understand the roles played by cytokines and growth factors during development and chemoprevention in NPC. Of particular interest are the observations that NPC patients showed a lower level of transforming growth factor-β1 (TGF-β1) in plasma, but a high level in tumor tissues and surrounding stroma compared to the healthy controls [6-9]. The TGF-β signaling pathway may play an important role in the carcinogenesis of NPC.

TGF-β belongs to a superfamily of structurally- and functionally-related cytokines, where the members of this family regulate a wide spectrum of cellular responses, including cell proliferation, differentiation, adhesion, migration and apoptosis [10]. It is now known that TGF-β is a cytokine that is a very potent inhibitor of cellular proliferation in normal cells. Evidence indicates that loss of the anti-proliferative responsiveness to TGF-β is a characteristic of many tumor cells [11-13], suggesting potential roles of TGF-β and substantial components of the TGF-β signal transduction pathway as tumor suppressors [14]. The Smad proteins are the principal intracellular components of the TGF-β signaling pathway, and it has been demonstrated that Smad proteins represent the most direct mediators for the transmission of signal from the cell surface in the nucleus [15]. Studies have shown that the expression of Smads is frequently altered in human cancers, for example, Smad4 has been found frequently inactivated in pancreatic [16,17], biliary[18], and colorectal tumors [19]. Increased expression of Smad6 and Smad7 has also been described in human pancreatic and prostate carcinomas [20,21], respectively.

The pathogenesis and the progression of numerous cancers have been attributed to the disruption of normal TGF-β signaling. However, the role of TGF-β signaling in the carcinogenesis of NPC is largely unknown, and it is not clear how NPC cells regulate TGF-β signaling in response to growth. Understanding the molecular mechanism underlying the TGF-β/Smad signaling pathway may provide a novel target for anticancer therapy. Herein, we described an *in vitro *study to examine TGF-β1 activity and ability to suppress tumor cell growth in a human NPC cell line. We conducted an analysis of the expression patterns of the TGF-β/Smad signaling pathway, its receptors and the intracellular Smads including Smad2, Smad3, Smad4 and Smad7. We also investigated the protein expression and subcellular localization of some components of Smads in response to the stimulation of TGF-β1 in the NPC cell lines.

## Materials and methods

### Cell lines, cell culture and treatment

The nasopharyngeal carcinoma cell lines (CNE2) and the immortalized nasopharyngeal epithelial cell line (NP69) were provided by the Biopharmaceutical Research and Development Center (Jinan University, Guangzhou, China), and cultured in Keratinocyte-SFM medium (Gibco, Carlsbad, CA) at 37°C in a humidified atmosphere of 5% CO_2_. Regarding the treatment of TGF-β1, the cells were plated at 5 × 10^3 ^per well in 96-well plate, and cultured in the presence of 10% FBS for 2 days. Then cells were washed and cultured with serum-free medium overnight, the next day, cells were treated with TGF-β1 at different concentrations in serum-free medium, and then continued to culture for 24 h, 48 h, 72 h, and 96 h, respectively.

### Cell growth response

To study the dose/time-effect response of CNE2 to TGF-β1, cells were plated at 5 × 10^3 ^per well in 96-well plate, and cultured in Keratinocyte-SFM medium for 24 h. Cells were washed and replaced in growth factors-free medium overnight and then treated with 0, 2.5, 5, 7.5, 10 and 12.5 ng/mL TGF-β1 in Keratinocyte-SFM medium. The status of cell growth was determined at 24, 48, 72 and 96 h, respectively, using Cell Counting Kit-8 (CCK-8) (Dojindo Laboratories China, Shanghai, China). CCK-8 solution was added into the plated cells at 10 μl/well, 4 h before each treatment and then the 96-well plate was swirled for 15 min. The spectrophotometrical absorbance of each sample was determined at 450 nm.

### Analysis of TGF-β receptors and Smads by RT- PCR

Cells were seeded at 1.6 × 10^5 ^cells per well into 6-well plate and cultured in Keratinocyte-SFM medium with growth factors for 24 h. Cells were washed and replaced with growth factors-free medium overnight, and then TGF-β1 was added (final concentration 10 ng/mL) for 3 h. Total RNA was isolated by using an RNA extraction kit and RNAex reagent (Huashun Biotechnology Co., Ltd., Shanghai, China) according to the manufacturer's instruction. Reverse transcription of 2 ng of total RNA was performed by using 20 units of AMV reverse transcriptase (BBI); 0.5 ng of oligo (dT) 12-18 primer; 0.5 mM each of dNTP and 20 units of RNase inhibitor in a total volume of 20 μL at 42°C for 60 min. The reaction was terminated by heating the mixture at 70°C for 10 min, and then was chilled on ice. After reverse transcription, PCR amplification was carried out in a volume of 20 μL containing 1× PCR reaction buffer, 0.2 mM dNTPs, 0.5 μM of sense and antisense primers (Table 1) and 1 unit Taq DNA polymerase, The reaction conditions included denaturation at 95°C for 30 s, annealing at 55°C for 30 s, and extension at 72°C for 45 s. The cycles were set at 30 cycles for TGF-β type II receptor (TβR-II), Smad2, Smad3, Smad4, Smad7 and 28 cycles for β-actin. Final extension was performed at 72°C for 10 min. PCR products were visualized by electrophoresis on a 2% agarose gel containing ethidium bromide as a fluorescent dye.

**Table 1:** PCR primer used in the experiment

Target mRNA	Primer sequence5'-3'	Product Size (bp)	GenBank Accession No
TβRII Sense	gca cgt tca gaa gtc ggt ta	493	D50683
Antisense	gcg gta gca gta gaa gat ga		
Smad2 Sense	aag aag tca gct ggt ggg t	246	AF027964
Antisense	gcc tgt tgt atc cca ctg a		
Smad3 Sense	cag aac gtc aac acc aagt	308	NM005902
Antisense	atg gaa tgg ctg tag tcg t		
Smad4 Sense	cca gga tca gta ggt gga at	243	U44378
Antisense	gtc taa agg ttg tgg gtc tg		
Smad7 Sense	gcc ctc tct gga tat ctt ct	320	AF015261
Antisense	gct gca taa act cgt ggt ca		
β-actin Sense	aca atg tgg ccg agg ctt t	260	M10277
Antisense	gca cga agg ctc atc att ca		

### Detection of the expression of Smads by Western blotting

Cells were seeded at 1.6 × 10^5 ^cells per well into 6-well plate, and cultured in Keratinocyte-SFM medium with growth factors for 24 h. Cells were washed and replaced with growth factor-free medium overnight, and then TGF-β1 was added (final concentration 10 ng/ml) for 3 h. The medium was removed and the cells were sonicated in lysis buffer containing 2% SDS, 10% glycerol, and 62.5 mM Tris (pH 7.0). Total proteins were collected by centrifuging at 12,000 × g at 4°C for 10 min, and separated by electrophoresis on a 12% sodium dodecyl sulfate polyacrylamide gel electrophoresis (SDS-PAGE) gel at 120 V, transferred to nitrocellulose membrane by blotting. After washing three times, the membranes were incubated with rabbit anti-Smad 2/3, rabbit anti-Smad 4, rabbit anti-Smad 7, rabbit anti-TGF-beta Receptor II, rabbit anti-Phospho-Smad2 (Ser245/250/255) antibodies (1:1000) (Cell Signaling Inc, Shanghai, China), and mouse anti-β-actin (Sigma, Shanghai, China) antibodies, respectively, for 2 h, then washed and incubated with secondary horseradish peroxide-conjugated antibody for 1 h. Antigen-antibody complexes were then visualized using an enhanced chemiluminescence kit (Amersham, Piscataway, NJ).

### Immunocytochemical analysis of TGF-β type II receptor and Smads

Cells were cultured on poly-L-lysine-coated chamber slides. As the cells confluence reached approximately 40%-50%, the medium was discarded and replaced with a serum-free Keratinocyte-SFM medium overnight. The next day, Keratinocyte-SFM medium containing 10 ng/mL TGF-β1 was added to treat the cells for 3 h, then washed with PBS for 5 min three times. The cells were fixed with 4% paraformaldehyde in PBS for 15 min at room temperature, and then were permeabilized by incubation in 0.1% Triton X-100 for 20 min at 37°C. Endogenous peroxidase was quenched with H_2_O_2 _in methanol (1:50). The cells were blocked with 5% BSA for 20 min at 37°C in a humid chamber. The primary antibodies were applied at a 1:100 dilution at 4°C overnight, the primary antibodies included anti-TβR II, anti-Smad2, anti-Smad3, anti-Smad4, and anti-Smad7 (Santa Cruz Biotechnology, Inc. Santa Cruz, CA). The biotinylated secondary antibody was applied for 20 min at room temperature in a humid chamber, and then the slides were rinsed in PBS for 5 min. Streptavidin biotin complex (SABC) was added to the slides and incubated in a humid chamber for 30 min at room temperature, and then rinsed in PBS for 5 min. The slides were applied with an aliquot of 3, 3'-Diaminobenzidine (DAB) to develop brown color. Counter-staining was performed with modified Mayer's hematoxylin for 10 s, washed with water for 10 min and mounted with resinous mounting medium after dehydration.

## Results

### CNE2 cells are insensitive to growth suppression by TGF-β1

**Figure 1:**
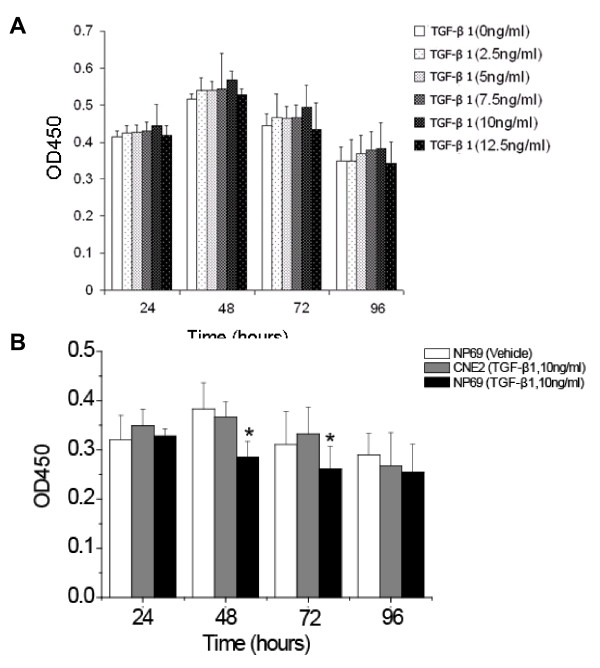
**Loss of the Growth-Inhibitory Effect of TGF-β1 on CNE2 cells**. CNE2 and/or NP69 cells were seeded in 96-well plate at 5 × 10^3 ^cells/well. (A) 2.5-12.5 ng/ml or (B) only 10 ng/mlTGFβ1 was added after 24, 48, 72, and 96 hours. Cell counting assay was used to indicate the degree of cell growth. Results were presented as the spectrophotometrical absorbance of cells treated with CCK-8 solution at the wavelength of 450 nm. * Statistically significant (P < 0.05, *t*-test) as compared with NP69 group. The values are expressed as means ± SD of six repeated experiments.

TGF-β1 is a potent growth inhibitor of epithelial cells. To test the response of human NPC cells to TGF-β1, we examined the growth pattern of CNE2 cells after TGF-β1 treatment. The rate of cell growth and the metabolic activity was indicated the degree of the growth suppression by TGF-β1 and a time course study regarding the growth suppression of CNE2 was performed. The data showed that the effect of growth suppression by TGF-β1 against CNE2 was not observed. Instead of suppression, CNE2 continued to grow after 24 h with TGF-β1 treatment at the various concentrations (2.5, 5, 7.5, 10, and 12.5 ng/ml), and reached a growth peak at 48 h after TGF-β1 treatment. Although TGF-β1 caused a slight increase in proliferation on CNE2 after TGF-β1 treatment by 48 h, no statistical significance was found compared to the untreated controls (Figure 1A). The insensitivity to TGF-β1 implied that the TGF-β1 signaling pathway could be abnormal in the CNE2 cells. To confirm the effect of growth suppression on the normal nasopharyngeal epithelial cells by TGF-β1, we performed the Cell Counting Kit-8 assay on the NP69 cells exposed to TGF-β1. Under the same experimental conditions, we used TGF-β1 at a concentration of 10 ng/ml because this concentration induced a high proliferation rate in the CNE2 cells among all time points tested. We monitored cell growth within 96 h after TGF-β1 treatment, and found that TGF-β1 did have the effect of growth suppression on NP69 cells. Adding TGF-β1 at a concentration of 10 ng/ml to the cell culture medium significantly reduced the viable cell number after 48 h, and the suppression rate of NP69 cells by TGF-β1 was statistically significant compared to the untreated NP69 cells (Figure 1B).

### TGF-β type II receptor and Smads in CNE2 cells

**Figure 2:**
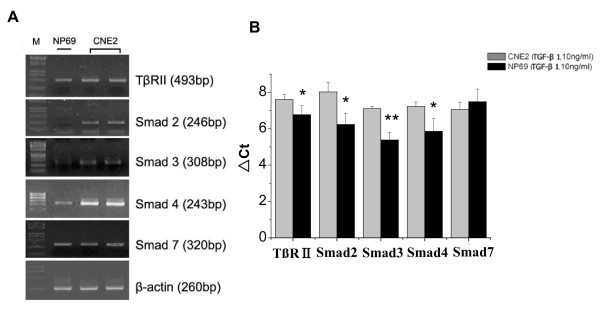
**The mRNA level of the TGF-β receptor II and the Smads in CNE2 and NP69 cells**. (A) Expression level of the TβRII, Smad 2, Smad 3, Smad 4, Smad 7 in CNE2 cells and NP69 cells by RT-PCR using specific primers. β-actin was used as a control and was further to normalize. (B) Bar diagram of the TβRII, Smad 2, Smad 3, Smad 4, Smad 7 mRNA level from densitometric measurement of three real-time quantitative PCR from three separate treatments. * Statistically significant (P < 0.05, *t*-test) as compared with NP69 group.** Statistically significant (P < 0.01, *t*-test) as compared with NP69 group.

**Figure 3:**
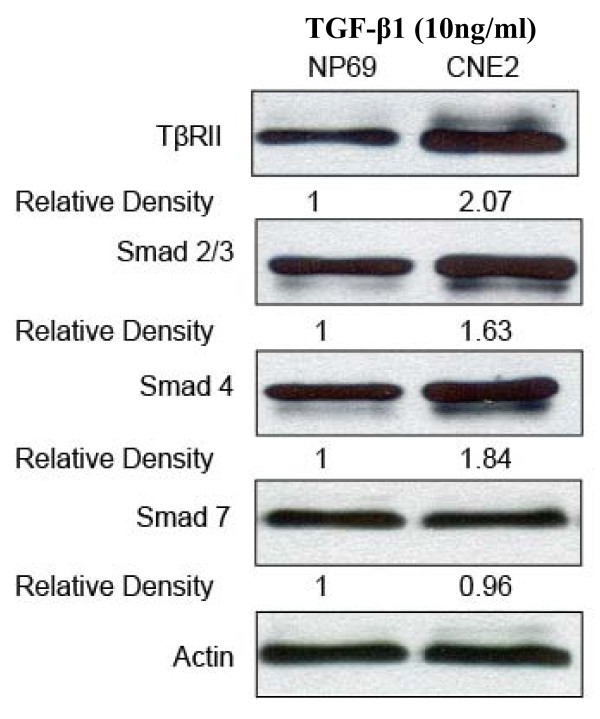
**The expression of the TGF-β receptor II and the Smads in CNE2 and NP69 cells**. Expression level of the TβRII, Smad 2, Smad 3, Smad 4, Smad 7 in CNE2 cells and NP69 cells by western blot. Actin was used as a protein loading control and was further to normalize. Relative density was account between NP69 and CNE2 group.

**Figure 4:**
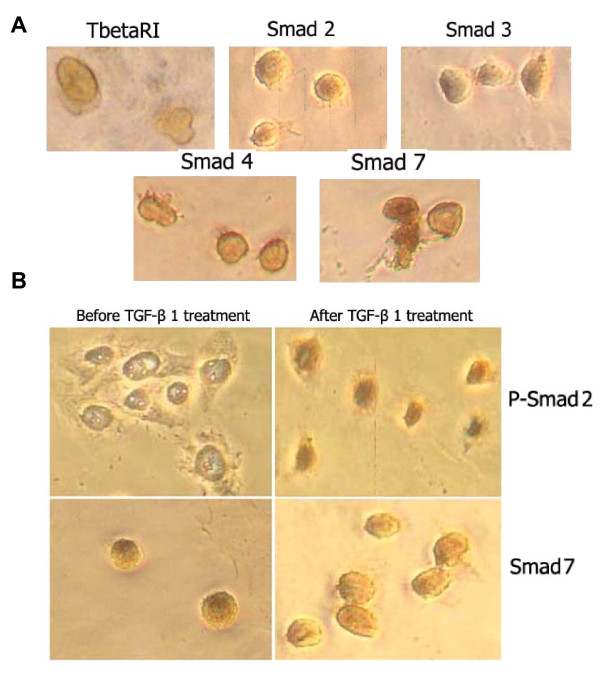
**Localization of expression of the TβR-II, Smad2, Smad3, Smad4, Smad7 and phosphorylated Smad2 in CNE2 cells**. (A) The TβR-II was located mainly in the cell membrane, and positive staining Smad2, Smad3, Smad4, was found in regions of both cytoplasm and nucleus, while the staining of Smad7 was mainly in the area of nucleus. (B) Phosphorylated Smad2 was undetectable in CNE2 cells without TGF-β1, after stimulation with TGF-β1, phosphorylated Smad2 could be detected in the cytoplasm of CNE2 cells, while Smad7 located originally in nuclear without TGF-β1, and it could be detected in the cytoplasm after stimulation of TGF-β1.

To investigate alterations of the TGF-β/Smad signaling pathway in CNE2 cells, the TGF-β type II receptor (TβR-II) and the TGF-β/Smad signaling components-Smads signal transduction were explored at both mRNA level and protein level by real time RT-PCR, using specific primers according to GenBank database sequences, western blotting and immunocytochemical analysis, respectively. First, we investigated TβR-II mRNA expression which is an upstream signaling partner of the TGF-β/Smad signaling pathway, while the normal nasopharyngeal epithelial cells were used as control. Under the same culture conditions, we found that TβR-II was significantly up-regulated in CNE2 cells compared to the levels observed in NP69 cells. We further evaluated the Smads which are the principal intracellular components of the TGF-β signaling pathway, and the results showed that Smad2, Smad3 and Smad4 mRNA all increased significantly in CNE2 cells compared to the levels observed in NP69 cells. However, the mRNA level of smad7, known as an inhibitory Smad, remained at same level as that observed for the normal nasopharyngeal cells (Figure 2A, 2B). To investigate the protein expression of the TβR-II receptor and Smads, western blotting was performed in NP69 and CNE2 cells. We found that Smad2, Smad3, Smad4 and TβR-II were also up-regulated in protein levels, but Smad7 protein level were no different compared to that observed in NP69 cells (Figure 3). To further localize the expression of the above signaling components in CNE2 cells, immunocytochemical staining was conducted. A positive staining of TβR-II was found in most CNE2 cells, and the cell membrane was the main localization of the protein. The positive staining of Smad2, Smad3 and Smad4 was found in regions of both the cytoplasm and nucleus, while the staining of Smad7 was mainly in the nucleus (Figure 4A).

### TGF-β1 inducing activation and translocation of Smad proteins in NPC cells

To determine whether Smad is activated and translocated in response to TGF-β1 stimulation in CNE2 cells, we assessed the subcellular distribution of the phosphorylated (activated) Smad2/3 by immunocytochemistry staining. No phosphorylated Smad2/3 staining was exhibited in CNE2 cells without TGF-β1 stimulation, however, a very strong staining of phosphorylated Smad2/3 was found in regions of both the cytoplasm and nucleus of the CNE2 cells after TGF-β1 treatment compared to untreated cells. This result indicated that Smad2 was phosphorylated and activated after TGF-β1 stimulation. Furthermore, we investigated the inhibitory Smad-Smad 7 protein in response to TGF-β1 stimulation in CNE2 cells. The results indicated that the positive staining of Smad 7 initially was localized in the region of the nucleus before TGF-β1 treatment. However, positive staining of Smad 7 was observed in the cytoplasm after TGF-β1 treatment, which implied that Smad 7 translocated from the nucleus to the cytoplasm in response to the TGF-β1 stimulation (Figure 4B).

## Discussion

TGF-β1 is a very potent inhibitor of many epithelial tumors, however, the role of TGF-β1 in nasopharyngeal Carcinoma progression is ambiguous. In the present study herein, we demonstrated for the first time that CNE2 cells have lost the sensitivity to growth suppression by TGF-β1 (Figure 1). Interestingly, rather than a defective TGF-β/Smad signaling pathway which leads to a loss of response to the growth suppression effect of TGF-β1, our results indicate that the TGF-β/Smad signaling is functional in the CNE2 cell after treatment TGF-β1. The TβR-II is expressed normally, while Smads 2, Smads 3, Smads 4 are significantly increased at the mRNA level and the protein level compared to the levels observed in the normal nasopharyngeal epithelial cells (Figure 2, 3). The mRNA and protein expression of Smad7 remains unchanged in the CNE2 cells. Immunocytochemistry demonstrated that the transmembrane receptor TβR-II and the intracellular component Smads are also detectable (Figure 4A), where pretreatment of CNE2 cells with TGF-β1 causes activation of the Smad 2 protein, and the inhibitory Smad 7 translocates from the nucleus into the cytoplasm (Figure 4B).

It has been demonstrated that TGF-β1 binds to the TβR-II and then phosphorylated the Smad2 and Smad3 after stimulation by TGF-β, and subsequent form hetero-oligomers with the common mediator Smad4. The formed Smad complex then translocates into the nucleus to regulate the expression of downstream genes [22,23]. Studies have demonstrated that loss of the TGF-β/Smad signaling function including defects in TGF-β receptors and/or downstream signal molecular Smad proteins is associated with tumor progression, and specific defects in this signalling pathway has been found in many cancers, including pancreatic, breast, ovarian, colorectal, liver, prostate cancer, leukemia, etc. [24-30]. Disruption of this TGF-β/Smad signaling cascade is considered an important mechanism by which tumor cells can escape growth suppression, and many cancer cells lose responsiveness to TGF-β-induced growth inhibition [10]. Our results indicate that CNE2 cells are not sensitive to the effect of growth suppression by TGF-β1 (Figure 1), suggesting that CNE2 cells may eliminate a critical negative control of TGF-β1 signaling. To assess whether the TGF-β/Smad signaling pathway in CNE2 cells changed or not, we investigated the expression of the components in the TGF-β/Smad signaling pathway, including TβR-II, Smad2, Smad3, Smad4, and Smad7. The results showed that all of these components of the TGF-β/Smad signaling pathway were expressed, and the mRNA expression of Smad2, Smad3 and Smad4 markedly increased (Figure 3). However the mRNA expression of the transmembrane receptor-TβR-II and Smad7 which participates in negative control of TGF-β1/Smad signaling pathway were left unchanged compared with normal nasopharyngeal epithelial cells (Figure 2). We further tested whether TGF-β1 can cause activation of Smad2 because phosphorylated activation of Smad2 is a key step in TGF-β1/Smad signaling for the induction expression of downstream molecules, and the results showed that exposure of cells to TGF-β1 did induced the phosphorylation of smad2 in CNE2 cells (Figure 4B), and TGF-β1 can also induce the translocation of smad7 from nucleus to cytoplasm (Figure 4B), suggesting that the TGF-β1/Smad signaling transduction is functional.

Although our results are different from the reports that the TGF-β/Smad signaling pathway is defective in the cancer cells, it is possible that the TGF-β/Smad signaling transduction is functional but the growth of CNE2 cells themselves are not suppressed by TGF-β1. The reason could be as follows. First, hundreds of genes are activated or repressed in response to TGF-β1 ligand stimulation, and the particular array of genes is cell-type- and condition-specific because the transcription factors utilized are cell-type- and condition-specific [31,32]. TGF-β1 has widely varying and divergent cellular effects although it uses an identical signaling system. Hence, it could be that CNE2 cells achieve resistance to the tumor-suppressor effect of TGF-β1, but remain responsive to the tumor-promoter effects of TGF-β1 via selective alterations of this signaling pathway. Second, TGF-β1 has a broad and multifunctional role because of this intricate system of components. Besides Smad-mediated transcription, TGF-β1 could also activate other signaling cascades, including MAPK, Erk, JNK and other yet-to-be-determined Smad-independent pathways [33]. Although this convergence of Smad-dependent and Smad-independent pathways in TGF-β family signaling can result in cooperativity, these pathways may also counteract each other, thereby enabling CNE2 cells to escape the tumor-suppressor effects of TGF-β1 and becoming resistant to TGF-β1-induced growth inhibition. Third, although it is generally accepted that TGF-β1 acts as a tumor suppressor through its ability to induce growth arrest at early stages, TGF-β1 can also act as a tumor promoter. Numerous studies have demonstrated that most cancer cells secrete larger amounts of TGF-β1 than their normal cell counterparts, and this overexpression is strongest in the most advanced stages of malignancies including nasopharyngeal carcinoma [6,7]. These malignancies can subvert TGF-β1 for their own purposes of survival, promoting angiogenesis, cell spreading, immunosuppression, tumor cell invasion and metastasis at late stages of tumorigenesis [34-37]. The CNE2 cell is a late-phage differentiation NPC cell line, so TGF-β1 is likely to serve as a tumor promoter rather than a tumor suppressor in CNE2 cells. Lastly, although the mechanism by which TGF-β1 switches its growth inhibitory effect into growth stimulatory effect is not well understood, TGF-β1 has been shown to increase the production of several mitogenic growth factors including TGF-α, FGF and EGF [38].

In addition, prolonged experimental exposure to high levels of TGF-β has been demonstrated to promote neoplastic transformation of intestinal epithelial cells, and TGF-β1 stimulates the proliferation and invasion of poorly differentiated and metastatic colon cancer cells [39,40]. Currently, less is known regarding the role of TGF-β1 and the TGF-β/Smad signaling pathway in the CNE2 cell, however, one study by using DNA microarray analysis demonstrates that the genes of TβR-I and TβR-II are upregulated in CNE2 cells [41], which is consistent with the our observation that TβR-II is expressed normally in CNE2 cells (Figure 2, 3).

In summary, an important issue addressed in this study is that CNE2 cells are not sensitive to growth suppression by TGF-β, but the TGF-β/Smad signaling transduction is functional. Further work is necessary to delineate a more detailed spectrum of the TGF-β/Smad signaling pathway, as well as understanding its crosstalk with other signaling pathways in CNE2 cells. By analogy to the situation in nasopharyngeal carcinoma, the components of the TGF-β/Smad signaling pathway may be a new target in the chemoprevention and chemotherapy of nasopharyngeal carcinoma.

## Competing interests

The authors declare that they have no competing interests.

## Authors' contributions

YDH and XKL designed the experiments. JX and QX carried out most of experiments and drafted the manuscript. YCX and ZJS carried out the immunocytochemistry. ZFH, QHZ and YT participated in statistical analysis and interpretation of data. All authors read and approved the final manuscript.
